# Research for VISION 2020

**Published:** 2010-12

**Authors:** Peter Ackland

**Affiliations:** Chief Executive, International Agency for the Prevention of Blindness (IAPB), London School of Hygiene and Tropical Medicine, Keppel Street, London WC1E7HT, UK.

**Figure:**
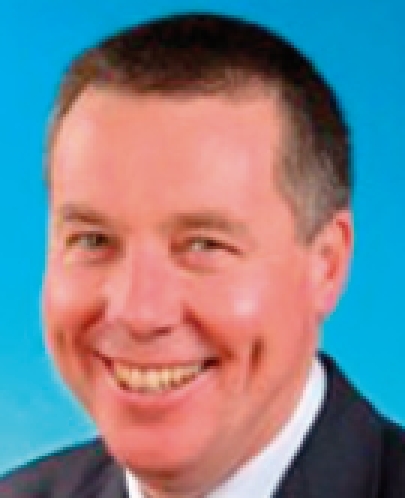


We need good quality information to be able to carry out our eye care programmes in support of VISION 2020, to measure (and improve) our performance, and to advocate for the resources and support we need to succeed. Much of this information can be collected, analysed, and used as part of our daily work, as many of the articles in this issue show.

However, many of our questions can only be answered by dedicated research. With the outcomes of such a large amount of eye care research available internationally, we can be forgiven for thinking that no 5 more research is necessary. Unfortunately, this is not so.

For example:

- Treatment paradigms established by research in resource-rich areas may not be an appropriate basis for delivering care in resource-poor areas. We need to test them and see what works best.
- Health systems research, critical to inform our programmes and policies, is currently under-prioritised and under-resourced, and will not take place without more support.
- New evidence is needed to help us plan eye care because the patterns of eye disease are changing constantly, both due to an ageing population and due to changing lifestyles.

**Figure:**
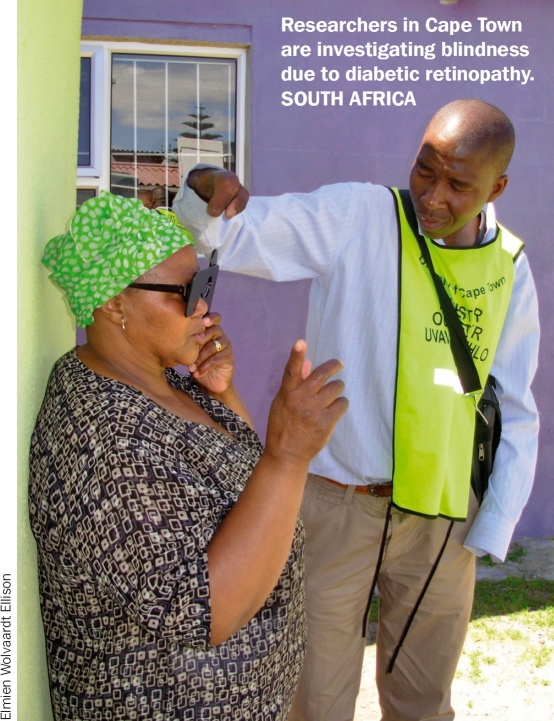
Researchers in Cape Town are investigating blindness due to diabetic retinopathy. SOUTH AFRICA

Elmien Wolvaardt Ellison

In addition, even when the appropriate research is available, there tends to be a gap between existing research and the actual use of evidence in the planning of blindness prevention programmes in the field; this is because research is often not easily accessible or not reported in a way that enables translation into actions.

Good research cannot take place without skilled staff and sufficient funding, but research often has to compete with eye care programmes for these resources. Therefore, we should be able to explain why we need to do research, why it is important, and how the outcomes of the research will support VISION 2020.

In September 2010, IAPB and the International Council for Ophthalmology jointly convened a workshop at the Lions Aravind Institute for Community Ophthalmology (LAICO) in Madurai, India, to consider research priorities and related issues. More than thirty representatives from different parts of the world and from a variety of research and programme backgrounds came together to generate an exciting new research agenda for the future.

**Priority list of global research themes**

**Diabetic retinopathy (DR)**

- Research to develop and test new paradigms for earlier treatment of DR relevant to resource-poor settings
- Research to develop and test interventions and systems effective in promoting and monitoring lifelong adherence to treatment of diabetes and diabetic eye disease within the non-communicable disease (NCD) framework

**Open-angle and angle closure glaucoma**

Research to develop low-cost and effective modalities and systems for identifying, treating, and monitoring glaucoma as well as promoting adherence to care in resource-poor settings

**Childhood blindness**

Research to demonstrate and evaluate a model for populations of up to 10 million at all levels of care which is comprehensive and integrated into child health programmes (includes evaluation of interventions to improve referral, uptake, coverage, and follow-up)

**Low vision**

Research to evaluate models and content for low vision care across the life spectrum as part of comprehensive health services in resource-poor settings

**Advocacy/impact**

Research to demonstrate and disseminate the economic, social, and quality of life benefits of eye care to individuals and societies

**Health systems**

- Research on the governance and structures within strengthened national health care and education systems necessary to optimise the delivery of, and demand for, cataract, trachoma, and refractive error services, leading to elimination of blindness and visual impairment from these conditions
- Research on the governance and structures within strengthened national health care and educational systems necessary to optimise delivery of, and demand for, comprehensive eye care services across the life spectrum

**Primary health**

Research on how to create and strengthen the systems for, and determine the benefits of, integrating primary eye health into primary health care and community development approaches

**Planning and monitoring progress**

Research to develop and test indicators and information systems to monitor eye care service outcomes at the programme, local, national, and regional levels, as part of integrated health management information systems

There were four main outcomes from the workshop:

1. A priority list of global research themes was determined - see page 43. These global priorities were underpinned with more detailed regional priorities and research needs for advocacy and health system strengthening.
2. A strong desire to invest in the capacity building of research institutions based in low- and middle-income countries and the recommendation that IAPB seek funding to promote this.
3. The need to ‘translate’ research -this means not only making it accessible and available to the people who need it, but also making research findings more easily understood by programme managers and policy makers and trying to break down the special language beloved of academia.
4. The creation of an IAPB research work group that will drive forward the ideas and recommendations from the workshop and also promote collaboration amongst IAPB members to support research work.

Delegates used the criteria listed below to set research priorities. These can be adapted to your own setting if you have to make decisions about allocating limited resources for research on a local, district, or national level.

- What is the likelihood that this research would have a major impact on reducing avoidable blindness by 2020?
- What is the likelihood that this research would improve our capacity to plan and deliver services?
- What is the likelihood that this research would contribute to greater resources being available for eye care services (e.g., evidence can be used for advocacy)?
- What is the likelihood that the impact of this research would lead to more equitable health outcomes across the region (e.g., research could help all segments of society, not just the privileged)?
- What is the likelihood of this study being designed and carried out to make a difference by 2020?

You can find a copy of the workshop report on the VISION 2020 website: **www.v2020.org**

